# Novel Transcriptional Mechanisms for Regulating Metabolism by Thyroid Hormone

**DOI:** 10.3390/ijms19103284

**Published:** 2018-10-22

**Authors:** Brijesh Kumar Singh, Rohit Anthony Sinha, Paul Michael Yen

**Affiliations:** 1 Laboratory of Hormonal Regulation, Cardiovascular and Metabolic Disorders Program, Duke-NUS Medical School, Singapore 169857, Singapore; 2 Department of Endocrinology, Sanjay Gandhi Post Graduate Institute of Medical Sciences, Raebareli Road, Lucknow 226014, Uttar Pradesh, India; anthony.rohit@gmail.com; 3 Division of Endocrinology, Metabolism, and Nutrition, Department of Medicine, Duke Molecular Physiology Institute, Duke University School of Medicine, Durham, NC 27710, USA

**Keywords:** thyroid hormone, gene transcription, metabolism, SIRT1, estrogen-related receptor alpha (ERRα), forkhead box protein O1 (FOXO1), microRNAs, non-alcoholic fatty liver disease (NAFLD), lipid metabolism, microRNAs (miRs)

## Abstract

The thyroid hormone plays a key role in energy and nutrient metabolisms in many tissues and regulates the transcription of key genes in metabolic pathways. It has long been believed that thyroid hormones (THs) exerted their effects primarily by binding to nuclear TH receptors (THRs) that are associated with conserved thyroid hormone response elements (TREs) located on the promoters of target genes. However, recent transcriptome and ChIP-Seq studies have challenged this conventional view as discordance was observed between TH-responsive genes and THR binding to DNA. While THR association with other transcription factors bound to DNA, TH activation of THRs to mediate effects that do not involve DNA-binding, or TH binding to proteins other than THRs have been invoked as potential mechanisms to explain this discrepancy, it appears that additional novel mechanisms may enable TH to regulate the mRNA expression. These include activation of transcription factors by SIRT1 via metabolic actions by TH, the post-translational modification of THR, the THR co-regulation of transcription with other nuclear receptors and transcription factors, and the microRNA (miR) control of RNA transcript expression to encode proteins involved in the cellular metabolism. Together, these novel mechanisms enlarge and diversify the panoply of metabolic genes that can be regulated by TH.

## 1. Introduction

Thyroid hormones (THs; triiodothyronine/T_3_ and thyroxine/T_4_) are critical regulators of the cellular and physiological metabolism. T_4_ is the major form in circulation and is converted to the more biologically active T_3_ in many tissues. THs can positively or negatively regulate the transcription of anabolic and/or catabolic gene subsets that affect energy homeostasis and metabolism [1]. For many years, THs were thought to control metabolism by directly regulating target gene transcription via the thyroid hormone receptor (THR), binding to thyroid hormone response elements (TREs) located on the promoters of target genes. However, only a limited number of transcriptionally-regulated genes have been identified which have direct THR binding to the conserved hexamer nucleotide sequences that comprise TREs [2,3]. Furthermore, it appears that TH can activate cellular pathways and physiological responses without THR binding to DNA or through the direct modulation of gene expression [4]. These discrepancies raise the possibility for alternative mechanisms of metabolic regulation by TH. In this review, we will describe some of these alternative mechanisms as well as examine new examples in which TH can modulate the transcription of subsets of target genes via the induction of metabolic changes that can lead to activation of other nuclear receptors/transcription factors, THR cross-talk with other cellular and nuclear proteins, and miRNAs [5,6,7,8,9,10,11].

## 2. Direct Regulation of Transcription by TH

THRs are ligand-dependent nuclear transcription factors that are generated from two different genes, *THRA* (NR1A1) and *THRB* (NR1A2) that encode the TRα and TRβ receptor isoforms, respectively. *THRA* and *THRB* are located on human chromosomes, 17 and 3. Interestingly, *THRA* generates two major isoforms (*THRA1* and *THRA2*) by alternative splicing to encode TRα1 and another isoform, TRα2, that has no T_3_-binding capacity [12,13]. *THRB* generates two major isoforms (*THRB1* and *THRB2*) from different transcriptional promoters to encode the TRβ1 and TRβ2 proteins [12,13]. The THR isoforms have variable spatial and tissue expression, particularly during development [3]. In addition to binding THs, THRs bind to TREs on the promoter regions of TH-regulated genes [3]. TREs usually contain two or more hexamer half-site sequences of AGGTCA arranged in tandem arrays that are typically separated by four nucleotides oriented in the same direction (AGGTCAnnnnAGGTCA) or in inverted palindromes [14,15]. THRs form multiple receptor complexes (monomers, homodimers, and heterodimers (with retinoid X receptors, RXRs)) but their precise role(s) may vary depending upon the ligand availability and gene context. In general, it is thought that unliganded THRs can bind as both homodimers and heterodimers to TREs, whereas liganded THRs bind primarily as heterodimers [12,13]. In the absence of TH, THRs bind to TREs and form a co-repressor complex with co-repressors (such as silencing mediator for retinoid and thyroid hormone receptors (SMRT)/nuclear receptor co-repressor (NCoR)) and histone deacetylases (HDACs) to deacetylate histone proteins, which, in turn, generate a more closed conformation of chromatin that represses transcription. For gene activation, the TH-bound heterodimer binds to TREs and interacts with a family of co-activators (steroid receptor co-activators (SRCs)) to recruit other co-activator proteins and histone acetyltransferases (HATs) such as such as P300/CBP-associated factor (PCAF/KAT2B) and CREB-binding protein (CBP)/p300, that alter the chromatin structure into a more open conformation. The subsequent recruitment of vitamin D receptor-interacting protein (DRIP)/thyroid hormone receptor-associated protein (TRAP)220 complex facilitates RNA polymerase II and general transcription factor binding to the promoter and activates gene transcription [16,17,18,19,20]. Ohba et. al. recently showed that acetylation of H3K9/K14 was associated with the acute stimulation of positively-target genes whereas H3K9 acetylation was not required for the chronic stimulation of gene expression [21]. These findings suggested that histone modifications may vary temporally among target genes. Although transcriptome analyses previously showed that THs can positively- and negatively-regulate the expression of similar numbers of target genes in several different tissues [12,13], the mechanism for the latter still is still not well understood but may involve THR recruitment of co-repressors to the TRE in the presence of TH [12,13]. The evidence supporting the foregoing general model for TH-mediated transcription of target genes has been reviewed extensively elsewhere [1,3,13,22,23,24,25,26]. A recent review of the nomenclature for different types of transcriptional regulation by TH has categorized them into 4 types with this classical type of TH action designated as Type 1 transcriptional regulation [23].

## 3. Major Alternative Types of Transcriptional Regulation

In addition to Type 1 regulation of transcriptional activity by THRs, there are alternative mechanisms for transcriptional regulation by TH. A recent consensus paper tried to categorize these alternative mechanisms, which are now referred to as Types 2–4 [23]. Briefly, these mechanisms attempted to account for TH regulation of transcription without the direct binding to TREs by THRs (as occurs in Type 1 transcriptional regulation). In Type 2 transcriptional regulation, THRs regulated target gene expression without the direct binding to DNA, and instead, bound to another protein or multi-protein complex in order to tether indirectly to the DNA. In Type 3 transcriptional regulation, THRs are not recruited to DNA to regulate transcription, and in Type 4 transcriptional regulation, TH can act independently from THRs by binding directly to other proteins. Some examples of these types of TH actions include:

*Type 2 transcriptional regulation*. Other modulator proteins or transcription factors can interact with TRs bound to TREs as well as vice versa with the latter referred to as Type 2 transcriptional regulation. Lin et al. [27] found that cyclin D1 (an oncogene) physically interacted with TRβ1 in a T_3_-independent manner to repress TRβ1′s silencing activity in an unliganded state and transcriptional activity in the liganded state. Yap et al. and Bhat et al. [28,29] showed that p53 (a tumour suppressor) physically interacted with THRβ1 and the latter’s DNA-binding domain to inhibit transcription activity by TRβ1. Interestingly, in a reciprocal manner, TRβ1-p53 dimerization led to an increased p53-DNA binding to p53 DNA binding elements in p53 target genes [30], demonstrating that TRβ1 also co-regulated the p53-mediated gene expression (Type 2 transcriptional regulation). Tardáguila M et al. [31] demonstrated that liganded THR directly interacts with Aurora kinase B and increased its kinase activity to displaces HP1β from the promoter region, thus preparing the chromatin for the transcriptional activation of TH regulated genes. Furthermore, liganded THRs also were shown to antagonize transcriptional activation by transforming growth factor beta (TGF-β)/SMAD through the reduced phosphorylation of SMADs and a direct interaction of the receptors with SMAD3 and SMAD4 [32]. 

*Type 3 transcriptional regulation*. Liganded TRβ associated with the PI3K-regulatory subunit p85α in the cytosol to activate phosphoinositide 3-kinase (PI3K), phosphorylate protein kinase B (PKB/AKT), and mammalian target of rapamycin (MTOR), and the latter’s substrate ribosomal protein S6 kinase beta-1 (p70S6K), to induce the hypoxia-Inducible Factor 1A (*HIF-1α*) transcription factor and the expression of its target genes, glucose transporter 1 (*GLUT1*), phosphofructokinase platelet (*PFKP*), and monocarboxylate transporter 4 (*MCT4*) [33,34]. Moreover, the THRB^PV/PV^ mutant bound significantly more to the PI3K-regulatory subunit p85 in the mutant TRβ (THRB^PV/PV^) mice than the wild-type mice [35]. This resulted in a greater increase in the PI3K kinase activity and activation of the PI3K-AKT-MTOR-p70(S6K) pathway in the cytoplasmic and nuclear compartments [35,36]. Furthermore, the THRB^PV^ mutant also bound to β-catenin, a transcriptional co-activator that regulates cell proliferation and cell survival, which was highly elevated in the thyroid tumours of THRB^PV/PV^ mice [37]. The interaction favoured the unliganded state of TRβ; hence, it was T_3_-dependent. The increased physical interaction between β-catenin and unliganded TRβ led to the activation of β-catenin-regulated downstream target genes. 

*Type 4 transcriptional receptor*. THs have been shown to bind to integrin αvβ3 membrane receptor proteins independent of THRs [38]. αvβ3 membrane protein originally was thought only to recognize and bind to extracellular matrix proteins (ECMs), so it was quite surprising when it was found that THs could bind to integrin near its Arg–Gly–Asp recognition site [39]. Integrin αvβ3 has two binding domains and no structural homology with nuclear THRs. The S1 domain exclusively recognizes T_3_ and activates PI3K via Src kinase, whereas, the S2 domain regulates mitogen-activated protein kinases MAPK1 and MAPK2 and binds both T_4_ and T_3_. Interestingly, each S1 and S2 domain mediates specific downstream effects, S1 directs Src and the PI3K-mediated trafficking of intact TRα from the cytoplasm to the nucleus and causes the transcription of *HIF1A*. In contrast, S2 activates MAPK1 and MAPK2, which then promotes the nuclear uptake of TRβ1 from the cytoplasm and leads to the proliferation of tumour cells [19,39,40]. Genes regulated by TH-binding to integrin αvβ3 include fibroblast growth factor 2 (*FGF2*), matrix metalloproteinase 2 (*MMP2*), *HIF1A*, and cyclooxygenase 2 (*COX2*), which have been associated with cancer development and angiogenesis [39,40]. TH binding to intracellular proteins have also been reported and these may impact transcription of downstream genes [12].

## 4. Additional Mechanisms of Transcriptional Regulation by TH

Type 1 transcriptional regulation is the predominant mechanism for TH-mediated regulation of transcription; however, the presence of cytosolic THRs has raised the possibilities for other THR interactions with cytoplasmic proteins [41]. Moreover, these interactions could initiate intracellular signalling as well as generate or remove post-translational modifications of THRs in the cytoplasm as well as in the nucleus [39,40]. One major post-translational modification is the phosphorylation of THRs, which occurs in both the cytoplasm and the nucleus. Phosphorylation of THR increases the THR heterodimerization with RXR and enhances the THR stability and target gene transcription [42,43,44]. However, transcriptional effects of phosphorylation still require THR binding to DNA so they should be considered a subset of Type 1 transcriptional regulation. Other types of post-transcriptional modifications such as acetylation and sumoylation have also been described, and appear to enhance the THR-mediated transcriptional activity [6,45,46]. Thus, post-translational modification of THRs may, in certain cases, be a prerequisite for Types 1–3 transcriptional regulation. 

*Combined DNA binding and interaction with other transcription factors (Types 1 and 2)*. TH and Sterol regulatory element-binding protein 1 (SREBP1) have cross-talk as many target genes involved in lipogenesis are co-regulated by both receptors. SREBP1 (transcription factor that regulates gene transcription of hepatic lipogenesis pathway) expression induced ATP binding cassette subfamily D member 2 (*ABCD2*) through an sterol regulatory element (SRE) that overlapped with a direct repeat (DR-4) element. Both TRα and TRβ bound to this motif and modulated the SREBP1-dependent transcriptional activation of the *ABCD2* gene [47,48]. Similar findings, as well as evidence for physical interaction, were observed for SREBP1 and TRα on the acetyl-coA carboxylase (*ACC*) gene promoter [49]. THR and liver X receptor (LXR; lipogenic transcription factor) also bound to nearby response elements in the carbohydrate-responsive element-binding protein (*ChREBP*) promoter and physically interacted to enhance *ChREBP* gene expression in the liver; however, this effect seemed to be tissue-specific since THR alone was sufficient to increase the *ChREBP* transcription in white adipose tissue [50].

*Secondary regulation of target gene expression through the transcriptional induction of transcription factor expression*. Direct transcriptional regulation of the transcription factor, kruppel-like factor 9 (*KLF9*), by TH stimulated the expression of several downstream target genes in human and mouse hepatic cells [51,52]. In particular, the TH induction of *KLF9* stimulated mouse deiodinase 1 (*DIO1*) expression via two CACCC sequences located on both sides of the hepatocyte nuclear factor (HNF)4α-response element, as HNF4α and KLF9, in conjunction with HNF4α and GATA binding protein 4 (GATA4), synergistically activated the mouse *DIO1* promoter [52]. THRs also cooperated with KLF9 to regulate hepatocyte proliferation, differentiation, and the early stages of organogenesis by regulating by co-regulating a large number of target genes [51].

TRβ1 regulated the LXRα expression in a ligand-dependent manner to regulate hepatic lipogenic pathways [53]. However, TH also induced *ChREBP* (which controls the activation of glucose-induced lipogenesis in the liver) as well as *SREBP1* expression, leading to an increased transcription of their downstream target genes. Both THR and LXR co-regulated the expression of *ChREBP*, as TH stimulated the *ChREBP* gene transcription maximally when THR was bound to the LXR-response element 2 (LXRE2), and LXR to LXRE1 [54]. Recently, it was shown that TH stimulated the uncoupling protein 1 (*UCP1*) gene transcription due to the TH-mediated induction of the *ChREBP* expression in brown adipose tissue [55]. We have considered these secondary gene regulations via primary induction of the gene expression of transcription factors by TH as an extension of the Type 1 transcriptional regulation.

*Non-specific downstream effects on gene expression mediated by TH*. Recently, DNA methyltransferase DNMT3a was shown to be a direct target of TH and suggested that it may be an evolutionarily conserved mechanism for modulating global changes in DNA methylation [56]. Consistently, Tu et al. later found 6 aberrantly methylated genes with differential expression in thyroid cancer: peroxisome proliferator-activated receptor gamma coactivator 1-alpha (*PGC1α*), CREB binding protein (*CREBBP*), *p300*, CD44 antigen homing function and Indian blood group system (*CD44*), secreted phosphoprotein 1 (*SPP1*), and matrix metallopeptidase 9 (*MMP9*) [57]. These effects also may be considered as extensions of Type 1 transcription. Additionally, TH post-transcriptionally regulated the mRNA stability of thyroid hormone-responsive SPOT 14 homolog (*THRSP/SPOT14*) mRNA [58,59]. The precise mechanism for this effect by TH currently is not known.

*Transcriptional regulation by TH metabolites*. T_3_ (the most active form of TH) was previously thought to be responsible for most, if not all, of the TH effects on the metabolism in the cell. However, there has been accumulating evidence suggesting that other THs and TH metabolites that were originally thought to be less active (T_4_), or even inert 3,5-diiodo-l-thyronine (T_2_), as well as 3-iodothyronamine (T_1_ AM), have significant biological activities [22]. Recently, it was shown that T_2_ administration prevented the induction of insulin resistance by high-fat diet feeding [60]. Moreover, T_2_ rapidly stimulated hepatic fatty acid oxidation, decreasing hepatic triglyceride levels, and improving the serum lipid profile. Interestingly, T_2_ could mediate these effects in the absence of TRβ and thus suggested that they may involve a Type 4 transcriptional mechanism. These T_2_ actions have also been associated with improvements in healthy ageing [61], and suggest that T_2_ may have beneficial physiological actions in humans [61]. T_4_ has been shown to interact with integrin on the cell membrane to activate janus kinase/signal transducers and activators of transcription (JAK/STAT) signalling [40]. T1Am often acts in an opposing manner as TH and can induce a hypometabolic state in rodents that is opposite to the effects of T_3_ and T_4_ [62]. Since they occur without binding to THR, these examples can be considered a special subset of Type 4, except that they involve TH metabolites.

*TH regulation of mitochondrial DNA*. Early studies by Segal et. al. demonstrated the TH rapidly increased the 2-deoxyglucose uptake in chick embryo heart cells even in the presence of cycloheximide (a translation inhibitor), removing any involvement of the genomic pathway [63,64,65]. Wrutniak showed that there may be TR isoforms localized within the mitochondria [38,39,41,44]. In this connection, THR variants produced by alternative splicing or transcribed using alternative promoters produce THR proteins that are localized within mitochondria [14]. Truncated isoforms of TRα1, with a molecular weight of 43 kDa (p48) and 28 kDa (p28), respectively, were reportedly associated with mitochondrial DNA, and upon TH activation, increased mitochondrial transcription and protein synthesis [66,67]. Mechanistically, this regulation of mitochondrial transcription of DNA resembles Type 1 and/or Type 2 transcriptional regulation except for its occurrence in the mitochondria rather than in the nucleus. Currently, it is not known conclusively whether THR or THR variants bind directly or indirectly to mitochondrial DNA.

## 5. New Mechanisms of Transcriptional Regulation by TH

Besides the previous types of mechanisms that we have described, we recently discovered two novel mechanisms of transcriptional regulation by TH that further increase and diversify the number of target genes regulated by TH.

*Metabolic regulation of transcription factors by TH: TH activation of SIRT1*. Sirtuin 1 (SIRT1) is metabolically regulated by the nicotinamide adenine dinucleotide (NAD^+^)-dependent redox-sensitive protein deacetylase [68]. SIRT1 has been shown to regulate several metabolic pathways including the lipid, cholesterol, and glucose metabolisms as well as mitochondrial activity [68]. Since TH has also been shown to regulate many of the same metabolic pathways as SIRT1 [2,3], it was speculated that there could be TH-SIRT1 cross-talk. In this connection, SIRT1 and TRβ1 were found to physically interact with each other, and SIRT1 enhanced the TH-responsiveness in hepatic cells [6,7]. Interestingly, the SIRT1 interaction with TRβ1 promoted the latter’s deacetylation in the presence of T_3_ and enhanced the ubiquitin-dependent TRβ1 turnover [6]. A co-operative interaction between PGC1α and TRβ1 also was required in order to achieve the maximal TH–stimulated expression of the carnitine palmitoyltransferase 1α (*CPT1A*) gene, which contains a *bona fide* TRE in the promoter [69]. Additionally, PGC1α was shown to be activated by TH via SIRT1-dependent deacetylation, and it mediated the TH-dependent activation of pyruvate dehydrogenase kinase 4 (*PDK4*) and *CPT1A* [7,70,71]. Thus, SIRT1 may play important roles in stimulating and terminating THR-mediated transcription by interacting with THR or deacetylating THR and/or PGC1α. 

We recently showed that the TH stimulation of oxidative phosphorylation led to the metabolic activation of SIRT1 by increasing the intracellular NAD^+^ concentration. SIRT1 deacetylated forkhead box (FOX)O1 transcription factor and stimulated the expression of the gluconeogenic genes: phosphoenolpyruvate carboxykinase (*PEPCK*) and glucose-6-phosphatase catalytic subunit (*G6PC*) [5]. Later analysis of hepatic FOXO1 and TRβ1 ChIP-Seq data identified a specific subset of TH-stimulated FOXO1 target genes that required co-regulation by FOXO1 and TH [9]. In this connection, TH activation of FOXO1 depended upon an increase in SIRT1-MTOR Complex 2 (MTORC2) interaction and rapamycin-insensitive companion of MTOR (RICTOR) deacetylation, which led to a decreased AKT and FOXO1 phosphorylation [9]. Interestingly, the TH metabolite, T_2_, has also been shown to rapidly stimulate hepatic fatty acid oxidation via the activation of SIRT1, leading to the deacetylation and activation of PGC1α and the inactivation of SREBP1c with increased transcription of genes involved in mitochondrial biogenesis and decreased transcription of genes involved lipogenesis [60]. Of note, T_2_ has shown protection against intracellular damage in diabetic nephropathy and non-alcoholic fatty liver disease (NAFLD) in a SIRT1-dependent manner [72]. While the stimulation of SIRT1 activity by TH may activate other transcription factors and/or nuclear hormone receptors, it is interesting to speculate that it may also decrease the expression of some target genes by directly deacetylating histones or co-activators such as CBP and p300, which are active in the acetylated state [73,74].

The SIRT1-TRβ1 interaction may be protective for reducing TH-mediated transcriptional activation when general transcription needs to be reduced during times of nutrient or energy stress [75]. Of note, increased hepatic SIRT1 protein and reduced serum TH levels were observed in mice fasted for 48 h. Physiological thyroxine replacement prevented the increase in the SIRT1 expression [75]. In this connection, it has been proposed that, in the absence of TH, unliganded TRβ may reduce the proteasomal degradation of SIRT1 to conserve cellular energy, as well as decrease the TH-activation of metabolic and mitochondrial genes [75,76]. In this mechanism, the metabolic consequences of TH action, as well as THR, may modulate SIRT1 signalling and affect the downstream pathways when the transcription factors are deacetylated by SIRT1. This mechanism may rely on Types 1 and 2 to increase the oxidative phosphorylation and increase intracellular NAD^+^, as well as Type 3 by virtue of the THR interaction with SIRT1 and by SIRT1’s effect on THR acetylation. 

*Secondary regulation of transcription by TH: TH induction of PGC1α/ERRα*. Although this mechanism might seem to be a subset of secondary transcription by TH, it is complicated by the role of the secondary (PGC1α) and tertiary (ERRα) transcription factors co-regulating genes that may also be partially regulated by THR. It is noteworthy that this cascade is mediated by the cross-talk of three different nuclear hormone receptors.

Recent ChIP-Seq analyses of THR with other DNA interacting proteins such as FOXO1 and ERRα have shown that the THRs stimulated binding of these proteins to the promoters of their target genes. Additionally, THRs bound to DNA sequences in the promoters of target genes that were different than TREs and also bound to non-promoter regions [3,5,6,7,10,26], suggesting that THR interacted with other transcription factors/nuclear proteins or chromatin via protein-protein interactions at these sites or were being stimulated by the expression or activation of other transcription factors. Thus, the TH regulation of transcription may be more complex than originally thought and may undergo temporal changes depending upon its ability to express secondary transcriptional regulators in addition to its own direct transcriptional regulation. In this connection, it is noteworthy that more than 90% of the hepatic genes that were positively- or negatively-regulated by TH were different in mice injected daily with T_3_ for 10 days than those seen in mice only 6 hours after one injection with T_3_ [21,77]. These findings suggested that there may be a secondary induction of other transcription factors that regulated the transcription of downstream target genes. A good example of this phenomenon is the TH regulation of ERRα. Recently, TRβ1 and ERRα ChIP-seq analysis showed that many genes involved in mitochondrial metabolic pathways including oxidative phosphorylation, tricarboxylic acid (TCA) cycle, and β-oxidation of fatty acids, were co-regulated by both TRβ1 and ERRα [10], and suggested that genes and pathways that originally were thought to be regulated directly by TH via Type 1 regulation of transcription were, in fact, co-regulated by ERRα. Indeed, TH increased the ERRα expression in a TRβ1-dependent manner through the primary induction of PGC1α (a bona fide ERRα partner and considered to be an ERRα “protein-ligand”). This led to PGC1α-ERRα dimerization and the increased transcription of target genes involved in mitochondrial biogenesis and function such as nuclear respiratory factor 1 (*NRF1*), transcription factor A, mitochondrial (*TFAM*), and *CPT1A* Thus, this pathway plays a critical role in mitochondrial biogenesis, activity, and autophagy (mitophagy, see below). Of note, SIRT1 also deacetylated and activated both PGC1α and ERRα [78]; therefore, SIRT1 activation by TH also may play an additional important role(s) in the activation of the TH-PGC1α-ERRα axis. 

We recently showed that TH-PGC1α-ERRα was required to induce the Unc-51 like autophagy activating kinase 1 (*ULK1*) (an important kinase in mitochondrial fission and mitophagy pathway) gene transcription by TH suggesting that TH induction of ERRα played a pivotal role in mitophagy, in addition to mitochondrial biogenesis and function. TH stimulation of oxidative phosphorylation and ROS may be another stimulus for mitophagy. The TH-dependent increase in ROS led to activation of calcium/calmodulin-dependent protein kinase kinase (CAMKK)2 to phosphorylate 5′ AMP-activated protein kinase (AMPK), which in turn, phosphorylated ULK1 and led to its mitochondrial recruitment and initiation of mitophagy [79]. ULK1 was shown to be a critical regulator of both dynamin-1-like protein (DRP1) and FUN14 domain containing (FUNDC)1 phosphorylation as DRP1-dependent mitochondrial fission was likely necessary for subsequent removal of damaged mitochondria by mitophagy [80,81]. In this connection, the TH induction of *ULK1* expression via PGC1α-ERRα increased the phosphorylation and activation of both DRP1 and FUNDC1 to promote mitochondrial fission and mitophagy, respectively [10]. Thus, the TH-PGC1α-ERRα pathway that regulates mitochondrial turnover can be considered an extension of Type 1 transcriptional regulation with the addition of tertiary transcription factors (ERRα) and nuclear hormone receptors serving as the primary, secondary, and tertiary mediators of transcription.

## 6. TH Regulation of MicroRNAs (miRs)

MicroRNAs (miRs) are short non-coding RNA molecules approximately 20–22 nucleotides in lngth that are involved in the post-transcriptional silencing of RNA [82]. The expression of 40 miRs and transcripts from 92 known genes were significantly different in the livers of hypothyroid mice when compared to euthyroid controls [83]. Careful examination using in vivo and in vitro models showed that the expression of two target genes involved in methionine/cysteine transport and glycerol metabolism— major urinary protein 1 (*MUP1*) and glycerol-3-phosphate dehydrogenase 2 (*GPD2*)—respectively, were regulated by TH via miR-206 [83]. These findings suggested that the induction of miRs might enable TH to regulate the transcript expression or protein translation of target genes. Serum miR206 expression was also reduced in patients with hyperthyroidism. Moreover, since the inhibition of the endogenous miR206 expression led to decreased intracellular triglycerides and cholesterol levels in hepatic cells after TH treatment, it is possible that miR206 may play a role in the altered hepatic lipid metabolism during different TH states in the liver [84]. Yap et al. also found that TH induced miR-181d expression, which then negatively regulated caudal type homeo box transcription factor 2 (*CDX2*) (a positive regulator of hepatocyte differentiation), also decreased the sterol O-acyltransferase 2 (*SOAT2*) (an enzyme that generates cholesterol esters that are packaged into LDL lipoproteins) expression. These findings suggested that miR-181d may play a significant role in the reduction of serum cholesterol by TH by decreasing LDL lipoproteins [85].

TH stimulated miR-21 and decreased the T-lymphoma invasion and metastasis-inducing protein 1 (*TIAM1*) expression in order to reduce the hepatoma cell migration and invasion [86]. TH also induced the miR-214-3p expression to decrease proto-oncogene serine/threonine-protein kinase 1 (*PIM1*) expression, cell proliferation, and hepatoma formation [87]. On the other hand, TH negatively regulated the mature miR-17 transcript expression to increase *MMP3* expression in hepatocellular carcinoma (HCC) [88]. In contrast, the miR-130b down-regulation in hepatoma samples increased the *THRA1* and interferon regulatory factor 1 (*IRF1*) gene expressions, as well as EMT-related genes, the phosphorylation of MTOR, and the STAT3 and AKT activation-pathways that increase the motility and invasion of hepatoma cells [89]. Furthermore, the THR knockout mice displayed altered levels of miRNAs that led to changes in the target gene expression in skin [90]. This type of transcriptional regulation involving the miRNA expression by TH and THRs can occur directly or indirectly (Types 1, 2, or 3) depending upon the mechanism for regulation of miRNA expression by THRs. The expressed miRNAs are then involved in the special post-transcriptional gene regulation of target genes. 

## 7. Future Directions and Conclusions

**Figure 1:**
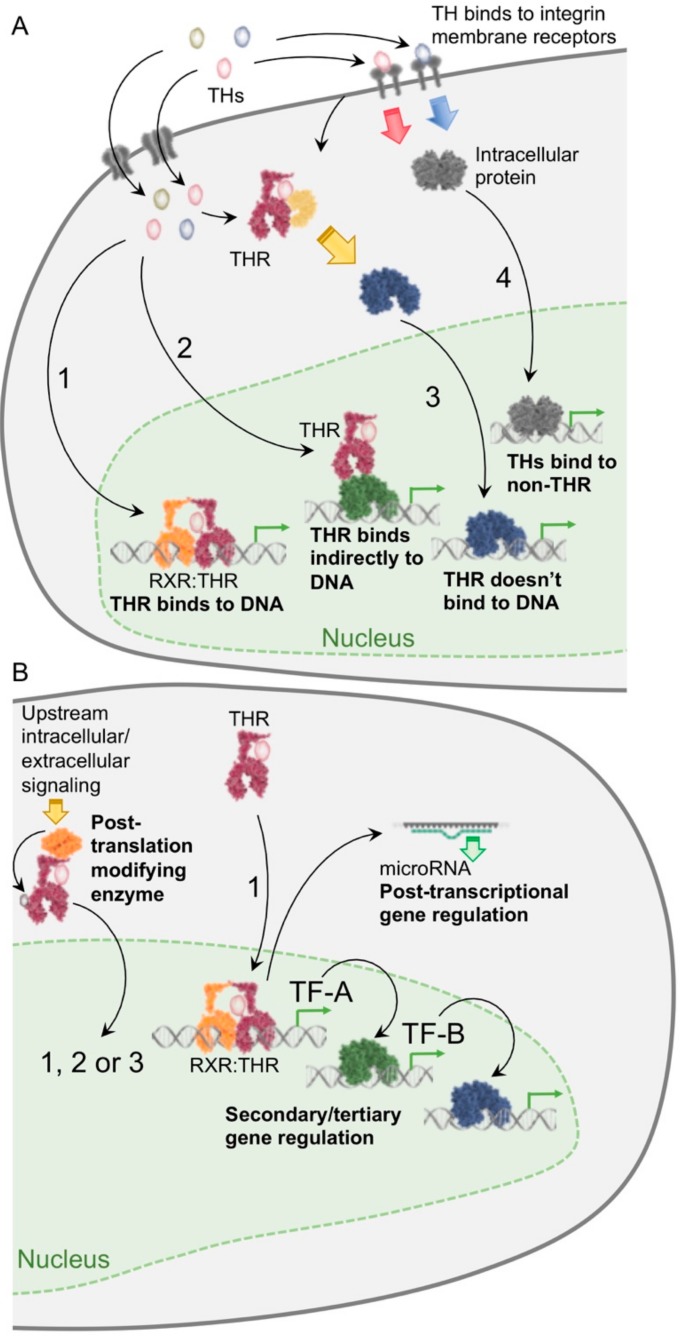
Transcriptional regulation by TH signaling. (**A**) *Four major categories of gene regulation by TH*. TH regulates gene transcription by a classical direct mechanism (Type 1 regulation), where liganded THRs heterodimerize with other nuclear receptors such as RXR, and recruit co-activators or co-repressors to directly activate or repress gene transcription, respectively. However, in Type 2 regulation, THRs do not bind directly to DNA and tether onto DNA by protein-protein interaction with other transcription factors. In Type 3 regulation, THRs do not require any binding to DNA and activate other transcription factors via non-genomic actions. In Type 4 regulation, TH binds to protein receptors other than THRs and activates gene transcription by other transcription factors. (**B**) *Alternative mechanisms for gene regulation by TH*. THR interaction with other proteins (such as SIRT1) leads to its post-translational modification and activation of gene transcription (subtypes of Type 1, 3). Metabolic activation of upstream regulator proteins such as SIRT1 by TH leads to the activation of other downstream transcription factors such as FOXO1 (subtype of Type 1). The TH-PGC1α-ERRα pathway that regulates mitochondrial turnover can be considered a special subset of Type 1 transcriptional regulation with the addition of secondary (PGC1α) and tertiary (ERRα) transcription factors and nuclear hormone receptors serving as the primary, secondary, and tertiary mediators of transcription. THR-mediated regulation of microRNAs shows post-transcriptional regulation and comes under Types 1, 2, or 3 depending on whether the mechanism involves the direct or indirect regulation of miRNA transcription.

Studies from the last two decades have shown that TH regulates the transcription of specific gene subsets during various metabolic conditions. Although the direct regulation of transcription by THRs has been extensively studied (Type 1 transcriptional regulation; Figure 1), there are also other mechanisms that regulate transcription ranging from the THR interaction with other transcription factors bound to response elements (Type 2 transcriptional regulation), the TH activation of THRs to mediate effects that do not involve DNA-binding (Type 3 transcriptional regulation), and the TH binding to proteins other than THRs (Type 4 transcriptional regulation) [23].

Recently, we and others have identified other transcriptional mechanisms such as the post-translational control of THRs (as a part of Type 1-3), TH-mediated induction of metabolic effects to activate other transcription factors by SIRT1, as well as the temporal and physical co-regulation of transcription by THRs in conjunction with other transcription factors (Type 1 and 2). Since SIRT1 activation and interaction with THR leads to the deacetylation of THR or other transcription factors, it might be considered a special subset of Type 3 transcriptional regulation. The THR-PGC1α-ERRα transcriptional pathway could be considered an extension of Type 1 for secondary transcriptional regulation after the primary induction of a transcription factor by TH. The post-transcriptional regulation by miRs to silence the mRNA expression of the target genes adds further complexity and fine-tuning of metabolic processes within the cell and can be considered as Types 1, 2, or 3 transcriptional regulation depending upon the mechanism, followed by the special post-transcriptional gene regulation. These different mechanisms may play important roles during acute vs. chronic responses to different nutrient conditions and hormonal states. It is also possible that different mechanisms (or combination of mechanisms) may predominate during different phases of target gene expression regulation by TH. These new mechanisms further increase the number and diversity of genes that can be directly and indirectly regulated by TH. Understanding the molecular mechanisms by which TH regulates the mRNA expression of target genes involved in nutrient and energy homeostasis will help us to better manage their pathophysiologic derangements in hypo- and hyperthyroidism. They may also enable us to employ TH and TH mimetics to treat metabolic conditions such as obesity, hypercholesterolemia, and non-alcoholic fatty liver disease.
